# miR-26a-5p Suppresses Wnt/*β*-Catenin Signaling Pathway by Inhibiting DNMT3A-Mediated SFRP1 Methylation and Inhibits Cancer Stem Cell-Like Properties of NSCLC

**DOI:** 10.1155/2022/7926483

**Published:** 2022-07-11

**Authors:** Jie Yu, Zhe Ge, Shunqiong Chen, Shaoying Li, Xin Zhang, Jie Hu, Wei Guo, Yan Wang

**Affiliations:** ^1^Department of Thoracocardiac Surgery, 920th Hospital of Joint Logistics Support Force of Chinese People's Liberation Army, Kunming, Yunnan 650032, China; ^2^Department of PCCM, 920th Hospital of Joint Logistics Support Force of Chinese People's Liberation Army, Kunming, Yunnan 650032, China; ^3^Laboratory of Molecular Cardiology, Department of Cardiology, The First Affiliated Hospital of Kunming Medical University, Kunming, Yunnan 650032, China

## Abstract

**Background:**

Lung cancer is a malignant cancer which results in the most cancer incidence and mortality worldwide. There is increasing evidence that the pattern of DNA methylation affects tumorigenesis and progression. However, the molecules and mechanisms regulating DNA methylation remain unclear.

**Methods:**

The expression of miR-26a-5p in NSCLC cell lines was detected by qPCR and verified in NSCLC tissues from TCGA using Limma R package. CCK-8 assay, plate clone formation assay, flow cytometry, and sphere formation assay were used to detect the cell proliferation, colony formation, cell cycle, and cancer stem cell- (CSC-) like property in NSCLC cell lines. The immunoblotting was used to detect the protein levels of DNMT3A, SFRP1, and Ki67. Global DNA methylation levels and DNA methylation levels of SFRP1 promoter were examined using ELISA and MSP-PCR assay, respectively. The distribution of *β*-catenin was examined using immunofluorescence (IF). Besides, xenograft mouse model was used to investigate the antitumor effects of miR-26a-5p in vivo. The pathology and protein levels were, respectively, detected by hematoxylin and eosin (H&E) and immunocytochemistry (IHC).

**Results:**

The expression of miR-26a-5p was downregulated in the tumor tissues comparted to adjacent normal tissues as well as NSCLC cell lines compared to normal lung epithelial cell (BEAS2B). The overexpression of miR-26a-5p inhibited cell proliferation, colony formation, CSC-like property, and arrested cell cycle at G1 phase. DNMT3A was a target of miR-26a-5p and upregulated DNA methylation on SFRP1 promoter. Mechanistically, miR-26a-5p repressed cell proliferation, colony formation, CSC-like property, and arrested cell cycle at G1 phase by binding DNMT3A to reduce DNA methylation levels of SFRP1 then upregulated SFRP1 expression. Moreover, miR-26a-5p exerted antitumor effects *in vivo*.

**Conclusion:**

Our results revealed that miR-26a-5p acted as a tumor suppressor through targeting DNMT3A to upregulate SFRP1 via reducing DNMT3A-dependent DNA methylation.

## 1. Introduction

Lung cancer is a malignant cancer which result in the most cancer incidence and mortality worldwide [1]. It has been estimated that lung cancer causes approximate 2.1 million new cases and 1.8 million deaths in 2018 [2]. In addition, an age-period-cohort analysis indicates that the incidence and mortality have increased in China during 1990-2017 [3]. Nonsmall cell lung cancer (NSCLC) is the largest subtype of lung cancer, which approximately 85% patients have been diagnosed as NSCLC [4]. Lung adenocarcinoma (LUAD) and lung squamous cell carcinoma (LUSC) are the most common subtypes of NSCLC [5]. The molecular heterogeneity remains leading cause of high recurrence and metastasis in NSCLC [6]. Despite rapid development of the therapeutic strategies for NSCLC in the past decades, the prognosis and survival of NSCLC remain depressed, which are due to the metastasis, chemoresistance, and recurrence [7, 8]. Therefore, the major strategy for NSCLC treatment is to explore the efficient target in NSCLC.

Cancer stem cells (CSCs) are a composition population of malignant cells in many myeloid leukemias and solider tumors with stem-cell like properties [9]. CSCs not only protect themselves from toxins and genotoxic stress via several mechanism to resistant to multiple therapeutic agents, including radiotherapy, chemotherapy, immune therapy, and other particular therapeutic agent [10], but also initiate tumor cells growth and metastasis [11]. CSCs have been identified according to the presence of the specific cell surface marker such as CD133, CD24, CD44, CD133, CD117, and aldehyde dehydrogenase 1A1 (ALDH1A1) [12, 13]. Increasing evidences have indicated that CSCs contribute to NSCLC tumor initiation, malignant progression, metastasis, and therapy resistance through modulating multiple mechanism, including TGF-*β*/TGF-*β*R signaling pathway and TGF-*β*/TGF-*β*R signaling pathway [14, 15]. In addition to heterogeneous properties, the differential genes, and dysregulation pathways, noncoding RNA and epigenetic alternation also affect tumor progression by modulating CSC properties.

MicroRNAs (miRNAs) are a group endogenous small noncoding RNAs that regulate target gene expression through binding with the 3′UTR of target gene to inhibit its expression in transcriptional or posttranscriptional levels [16]. Aberrant miRNA expression has been found in CSCs that provides the new insight and therapeutic target for tumor treatment [17]. For instance, miR-142-3p has been demonstrated to repress radio-resistance and breast cancer stem cell phenotypes [18]. miR-34a negative regulates CD44 to inhibit regeneration and metastasis through suppressing stemness prostate cancer [19]. Cancer stem cell-derived exosomes deliver miR-210 enhances gemcitabine resistance in pancreatic cancer [20]. However, the regulatory mechanism of miRNAs in NSCLC to modulate CSCs remains largely unknown.

In the past decades, the epigenetic regulation of DNA-templated processes including DNA methylation, histone modification, nucleosome remodeling, and chromatin remodeling emerges the pivotal function in tumorigenesis [21]. The methylation of the 5-carbon on cytosine residues (5mC) in CpG dinucleotides is the most common DNA modification and extensive modification on chromatin [22]. DNA methylation has been widely found in cancer based on the next-generation sequencing (NGS) technology [23]. DNA methylation at promoter suppresses expression of protein coding genes and various noncoding RNAs, and DNA methylation at gene body prevents aberrant transcription [24, 25]. DNA methyltransferase (DNMTs) activation represses gene expression by regulating the DNA methylation pattern that plays the therapeutic target for cancer therapy [26, 27]. However, the role and regulatory mechanisms of DNMTs in NSCLC remain unclear.

Here, we explored the role of miR-26a-5p in NSCLC based on The Cancer Genome Alas (TCGA) database and investigated whether the regulatory mechanism of miR-26a-5p in NSCLC associated with the DNA methylation of genes and CSC property regulation. In the present study, miR-26a-5p exerted tumor suppressor in NSCLC by targeting DNMT3A and then suppressing cancer stem cell-like properties in NSCLC by inactive Wnt/*β*-catenin signaling pathway.

## 2. Material and Methods

### 2.1. TCGA Data Acquiring and Processing

The level 3 of miRNA-seq data (RPM value), the level 3 RNA-seq data (FPKM value), and clinicopathological information were obtained from The Cancer Genome Alas (TCGA, https://portal.gdc.cancer.gov/). The miRNA profiling obtained from 567 samples of TCGA-LUAD include 521 LUAD tumor samples and 46 normal samples. Besides, the mRNA profiling collected from 594 samples of TCGA-LUAD, which include 535 LUAD tumor samples and 59 normal samples. After FPKM value of RNA-seq data was transferred into TPM value. The differentially expressed genes and miRNAs were screened using DESeq2 function of R package with log2|Fold change| > 2 and *P* value < 0.05.

### 2.2. Cell Culture, Treatment, and Transfection

The NSCLC cell lines (A549, HCC827, NCI-H23, and NCI-H1155) and normal lung epithelial cells (BEAS2B) were purchased from Shanghai cell Bank, China Academy of Sciences (Shanghai, China). All cells were cultured in DMEM medium (Invitrogen, Carlsbad, CA, USA) supplemented with 10% fetal bovine serum (FBS; Hyclone, South Logan, UT, USA) and 1% penicillin/streptomycin solution (Sigma-Aldrich, St. Louis, MO, USA). All cells were incubated in a humidified atmosphere at 37°C with 5% CO_2_.

The NSCLC cells were treated with the 5-Aza-2′-deoxycytidine (Aza; HY-10586, MedChem Express, Monmouth Junction, NJ, USA) for three days following the previous description [28]. 10,000 cells were seeded into 96-well plates and cultured at 37°C for 24 h. Then, 1 mL 5-Aza (0.5 *μ*M) was added into each well and incubated for 72 h for subsequent experimental analyses. Furthermore, the 10,000 cells were stimulated with 20 *μ*M HLY78, a specific Wnt/*β*-catenin pathway activator (HY-122816, MedChem Express, Monmouth Junction, NJ, USA), for 72 h for following experiments.

Overexpression or downregulation of miR-26a-5p was accomplished using the miRNA mimic and miRNA inhibitor. The DNMT3A was overexpressed using pcDNA3.1 (+) vector (Thermo Fisher Scientific, Waltham, MA, USA), and DNMT3A and SFRP1 were silenced using short hairpin RNA (shRNA) vector. All oligonucleotides of miRNA mimic and miRNA inhibitor and their negative control and all shRNA vectors were designed and synthesized from RIOBIO technological company (Guangzhou, China) and listed in Table S1. Oligonucleotides and vectors were transfected into cells using Lipofectamine 2000 (Invitrogen, Carlsbad, CA, USA) following the suggestion of manufacturer.

### 2.3. RNA Extraction and Quantitative PCR

Total RNA was extracted from tissues and cells using RNAiso plus (Takara, Dalian, China), and the miRNAs were extracted from tissues and cells using the RNAiso for Small RNA (Takara, Dalian, China) according to the manufacturer's instruction. RNA was reversed transcription into cDNA using PrimeScript™ RT reagent Kit with gDNA Eraser (Takara, Dalian, China) following the suggestion of manufacturer, and the qPCR was performed using a TB Green® Premix Ex Taq™ (Takara, Dalian, China) in a 7300 ABI Real-time PCR system. U6 was used for normalizing the miRNA and other gene expression. The relative expression of genes was calculated using the 2^-△△Ct^ methods in this study. The primers' sequences were listed as following, miR-26a-5p, F, 5′-TGGCCTCGTTCAAGTAATCCA-3′; R, 5′-CCCCGTGCAAGTAACCAAGA-3′. U6, F, 5′-CGGCACCATGTTGGTGGA-3′; R, 5′-AGGTACTTGATGGTGCTGCC-3′.

### 2.4. Cell Viability Analysis

Cell viability was measured using the cell count-kit 8 (CCK-8) assay. Briefly, after cells were transfection, 10, 000 cells were seeded into 96-well plates and incubated for 24 h, then 10 *μ*L CCK-8 solution was added into each well and incubated for 20 min. Finally, the absorbance of each well at 450 nm was measured using a microplate reader.

### 2.5. Colony Formation Assay

The cell colony ability was determined using the colony formation assay. After cell transfection, approximately 1000 cells were seeded into six-well plates and incubated for 14 days. Then, cells were fixed with methanol and stained with 0.1% crystal violet solution. After that, the colonies were observed, counted, and imaged under a microscope.

### 2.6. Cell Cycle Analysis

The cell cycle was analyzed using a flow cytometry (FCM). In brief, after cell transfection, approximately 2 × 10^5^ cells were plated onto 24-well plates and incubated for 24 h. Then, cells were harvested and stained with propidium iodide (PI, Beyotime, Shanghai, China), and then cell cycle was measured using FCM. Normally, the G1 phase cells were accumulated in the red area on the left side, and the S and G2 phase cells were enriched in the intermediate white and red area.

### 2.7. Sphere Formation Assay

The cancer cell stem-like property was demonstrated using a sphere formation assay. Generally, after transfection, 500 cells were seeded into six-well plates and cultured in the DMEM complete medium supplemented with 2% B27 (Sigma-Aldrich, St. Louis, MO, USA), 20 ng/mL EGF (Sigma-Aldrich, St. Louis, MO, USA), and 20 ng/mL bFGF (Sigma-Aldrich, St. Louis, MO, USA) for seven days. Then, the diameter of sphere with greater than 50 *μ*m was counted.

### 2.8. Immunoblotting Assay

Protein was isolated from tissues and cells using RIPA buffer (Beyotime, Shanghai, China) according to the manufacturer's instruction. Then, protein was separated using 10% SDS-PAGE gel, transferred onto PVDF membranes, and blocked with 5% nonfat milk powder. The membranes were then incubated with primary antibodies, including anti-OCT4 antibody (ab200834, 1 : 10000), anti-NANOG antibody (ab203919, 1 : 1000), anti-SOX2 antibody (ab92494, 1 : 2000), anti-DNMT3A antibody (ab188470, 1 : 2000), anti-SFRP1 antibody (ab267466, 1 : 1000), anti-*β*-catenin antibody (ab32572, 1 : 5000), anti-MYC antibody (ab32072, 1 : 1000), and anti-CCND1 antibody (ab40754, 1 : 2000) at 4°C overnight. Then, the bands were incubated with HRP-preadsorbed goat antirabbit IgG secondary antibody (ab7090, 1 : 500) at room temperature for 1 h. Finally, the bands were visualized using an enhanced chemiluminescent indicator (Bio-Red, Hercules, CA, USA). GAPDH was used as the internal reference protein. All antibodies in this study were purchased from Abcam (Cambridge, MA, USA).

### 2.9. Evaluation of the Cancer Stem Cell-Like Phenotype

The cancer stem cell-like cells were estimated using flow cytometry (FCM) by staining with surface markers including CD34, CD133, and ALDH1. Briefly, after transfection, 5 × 10^5^ cells were harvested and washed with PBS, then incubated with primary antibodies, including CD34 (ab81289, 1 : 50), CD133 (ab216323, 1 : 100), ALDH1 (ab52492, 1 : 20), and isotype control at 4°C for 30 min. Then, after washing with PBS for three times, cells were incubated with DyLight® Fluorochrome conjugated secondary antibodies (Abcam, Cambridge, MA, USA) at 4°C for 30 min. After that, cells were washed with PBS for three times and resuspended with 100 *μ*L PBS and sorted using FCM.

### 2.10. Dual-Luciferase Activity Assay

The binding relationship between miR-26a-5p and DNMT3A was determined a dual-luciferase activity assay. Briefly, the sequences of 3′UTR of DNMT3A were amplificated and inserted into the pmirGLO dual-luciferase vector to construct the wild type pmirGLO dual-luciferase vector (DNMT3A wt) and CUUG instead of GAAG to construct the mutant type pmirGLO dual-luciferase vector (DNMT3A mut). And then, cells were cotransfected with DNMT3A wt/mut vector and miR-26a-5p mimic or NC mimic and incubated for 48 h. Finally, the luciferase activity was detected using a dual-luciferase reporter gene assay (Promega, Madison, WI, USA).

### 2.11. Global DNA Methylation Level Analysis

The global DNA methylation levels were measured using the Global DNA methylation assay kit (5 methyl cytosine, colorimetric) (ab233486, Abcam, Cambridge, MA, USA) following the manufacturer's protocol. The absorbances at 450 nm of each well presented the amount of DNA methylation. In addition, the DNA methylation status of SFRP1 also was detected using a methylation-specific PCR (MSP) according to the previous described [29]. Generally, the genomic DNA was extracted from cells and purified using the TaKaRa MiniBEST Universal Genomic DNA Extraction Kit and (Takara, Dalian, China) following the manufacturer's instruction. Then, genomic DNA was modified by sodium bisulfite, and the modified genomic DNA subsequently was purified and recovered for following methylation-specific polymerase chain reaction (MSP-PCR). The modified genomic DNA samples were amplified using the specific primers for either the methylated (M) or unmethylated (U) DNA. DNA methylation was confirmed using human methylated/nonmethylated DNA stander (Shanghai Zeye Biotechnology, Shanghai, China) according to the manufacturer's protocol. The primers were designed based on the Laboratory of Molecular Medicine (The Li Lab) online database (http://www.urogene.org/index.html). A total five paired primers were synthesized and preamplified. Then, the methylated primer pairs (M) 5′-GTATTATTTGAGGTTAGGAGTTCGA-3′ and 5′-CTAAAATACAATAACGCTATCTCCG-3′, and the unmethylated primer pairs (U) 5′-GTATTATTTGAGGTTAGGAGTTTGA-3′ and 5′-AAAATACAATAACACTATCTCCACT-3′.

### 2.12. Immunofluorescence (IF) Analysis

The differential levels of *β*-catenin in subcellular location were determined using IF analysis. Generally, after cell transfection, the cells were fixed with methanol and incubated with primary antibeta catenin antibody (ab32572, 1 : 250, Abcam, Cambridge, MA, USA) at 4°C for overnight and incubated with HRP-preadsorbed goat anti-rabbit IgG (Alexa Fluor® 488) (ab150077, Abcam, Cambridge, MA, USA) for 1 h in the dark. After cells were counterstained with DAPI dye for 5 min, the cells were observed and photographed using a confocal microscope (Observer Z1 Confocal Spinning Disc V.2 Zeiss with live imaging chamber).

### 2.13. Animal Experiments and Pathological Analysis

Total eighteen 4-6-week-old female BALB/C mice were employed in this study. All animal experiments were approved by the Ethic Committee of Kunming Second People's Hospital and obeyed the Laboratory Animal Care. A549 cells were transfected with miR-26a-5p mimic or miR-26a-5p inhibitor to construct the stable overexpressed miR-26a-5p cells or knockdown miR-26a-5p cells. Then, the mice were randomly divided into three groups, the 1 × 10^7^ modified cells were transplanted into the flank of mice, and the mice were accepted with the untreated 1 × 10^7^ A549 cells. The mice were normally feed for 28 days, and the tumor size was observed and measured every 7 days until the mice were euthanized. After the mice were euthanized, blood was collected from heart, the serum was harvested for FCM analysis, and the tumors were separated for following experiments.

The pathological analysis was performed by hematoxylin and eosin (H&E) staining and immunocytochemistry (IHC). Briefly, the 4 *μ*m paraffin embedded section was deparaffined with xylene and rehydrated with a gradient concentration of ethanol. After heat induced epitope retrieval, the sections were stained with H&E solution (Beyotime, Shanghai, China) according the instruction of manufacturer. For IHC analysis, the sections were incubated with primary antibodies, including anti-Ki-67 antibody (ab16667, 1 : 200), anti-DNMT3A antibody (ab188470, 1 : 1000), and anti-SERP1 antibody (ab240023, 1 : 500) overnight at 4°C and then incubated with secondary antibody HRP-preadsorbed goat antirabbit IgG secondary antibody (ab7090, 1 : 500) at room temperature for 30 min. After that, the positive immunostaining signal was visualized using the 3,3′-diaminobenzidine (DAB) solution (Beyotime, Shanghai, China), cell nuclei were counterstained with hematoxylin. Then, the sections were observed and imaged under an inverse fluorescence microscope (Olympus, Tokyo, Japan).

### 2.14. Statistical Analysis

Statistical analysis was performed by GraphPad Prism version 9.0 (GraphPad Software, San Diego, CA, USA) in this study. Value was presented as mean ± standard deviation (SD), and comparison differences between two groups and among multiple groups were accomplished by unpaired *t*-test and one-way analysis of variance (ANOVA), respectively. The nonparametric test was performed by chi-square test. The overall survival was analyzed by the Kaplan-Meier plot using the log-rank test. *P* value < 0.05 was considered statistical significance.

## 3. Results

### 3.1. Downregulation of miR-26a-5p in NSCLC Tissues and Cell Lines

**Figure 1:**
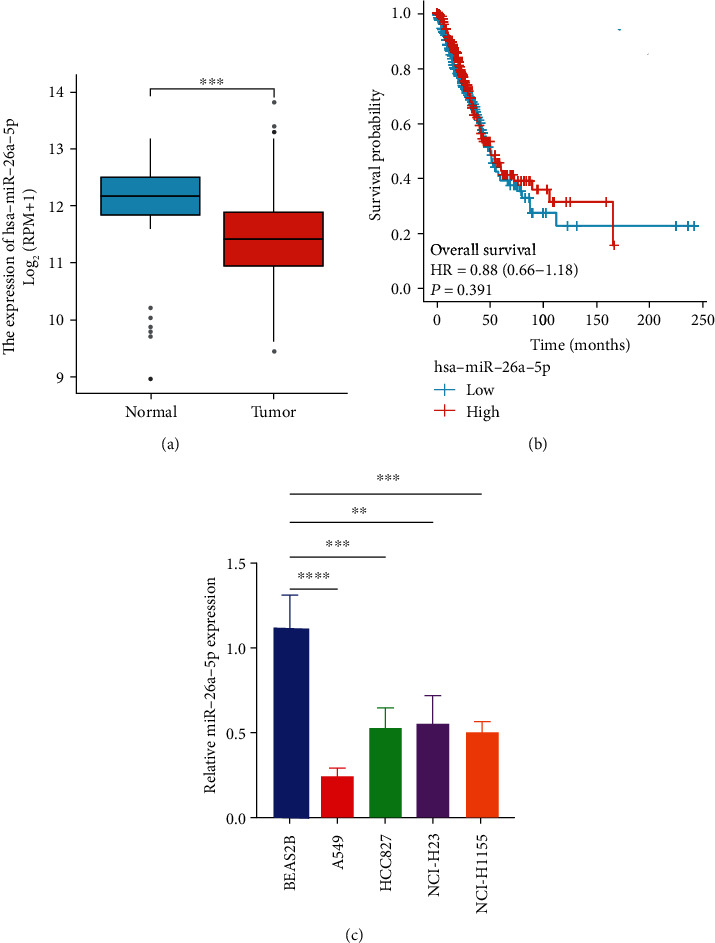
Downregulation of miR-26a-5p in NSCLC tissues and cell lines. (a) The expression of miR-26a-5p in LUAD tumor samples (*N* = 521) and normal samples (*N* = 46) based on TCGA database. (b) Overall survival between high miR-26a-5p expression group and low miR-26a-5p expression group was determined by Kaplan-Meier plot using log-rank test in TCGA database. (c) The expression of miR-26a-5p between NSCLC cell lines and BEAS2B was analyzed by qRT-PCR. ∗∗*P* < 0.01; ∗∗∗*P* < 0.001.

First, we demonstrated miR-26a-5p downregulated in LUAD tumor tissues compared to normal tissues based on TCGA data (Figure 1(a)). OS curve indicated high expression of miR-26a-5p associated with favorable survival rate (Figure 1(b)). We explored miR-26a-5p expression in NSCLC tissues and cell lines, and qPCR results indicated miR-26a-5p downregulated in NSCLC tissues compared to adjacent normal tissues as well as in NSCLC cell lines (A549, HCC827, NCI-H23, and NCI-H1155) compared to normal lung epithelial cells (BEAS2B) (Figure 1(c)). Also, we found that miR-26a-5p was correlated with the T stage of LUAD (Table S2). Above results suggested that miR-26a-5p acted as a tumor suppressor in NSCLC.

### 3.2. miR-26a-5p Suppresses Cell Viability and Stem Cell-Like Phenotype in NSCLC

**Figure 2:**
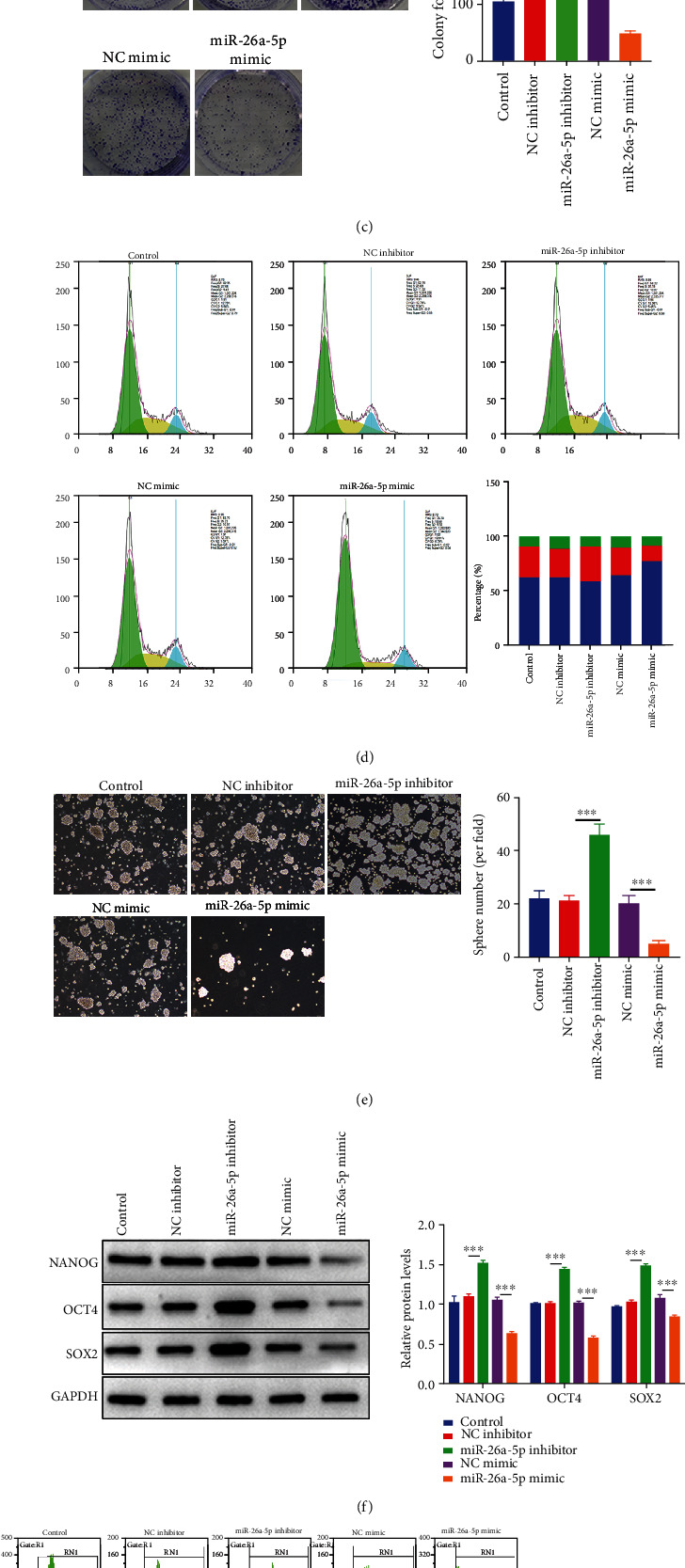
miR-26a-5p suppresses cell viability and stem cell-like phenotype in NSCLC. (a) The expression of miR-26a-5p was detected by qRT-PCR after miR-26a-5p upregulation/downregulation. (b) Cell viability was examined using CCK-8 assay after miR-26a-5p upregulation/downregulation. (c) Cell colony formation was determined by plate clone assay after miR-26a-5p upregulation/downregulation. (d) Cell cycle was detected by FCM after miR-26a-5p upregulation/downregulation. (e) CSC-like property was detected using sphere formation assay after miR-26a-5p upregulation/downregulation. (f) Protein levels of NANOG, OCT4, and SOX2 were determined by western blotting after miR-26a-5p upregulation/downregulation. (g) CD34, CD133, and ALDH1 positive cells were identified by FCM after miR-26a-5p upregulation/downregulation. Control group was used as the blank control. ∗∗∗*P* < 0.001.

We further detect the function of miR-26a-5p in NSCLC using gain and lose of function experiments. miR-26a-5p expression was upregulated and repressed by transfecting with miR-26a-5p mimic and miR-26a-5p inhibitor, respectively (Figure 2(a)). Then, we examined the cell viability using CCK-8 assay, and cell viability was repressed by miR-26a-5p overexpression but increased by miR-26a-5p inhibition (Figure 2(b)). In addition, the cell colony formation ability was inhibited by miR-26a-5p overexpression whereas elevated by miR-26a-5p inhibition (Figure 2(c)). Cell cycle was arrested at G1 phase by miR-26a-5p overexpression but the cell cycle was accomplished by miR-26a-5p inhibition (Figure 2(d)). Furthermore, we also investigated the effects of miR-26a-5p on cancer stem-cell like properties. The cancer cell sphere formation ability was repressed by miR-26a-5p overexpression and promoted by miR-26a-5p inhibition (Figure 2(e)). miR-26a-5p overexpression suppressed the protein levels of OCT4, NANOG, and SOX2, and embryonic stem cell markers are associated with cancer stem cells, while miR-26a-5p inhibition promoted the expression of these markers (Figure 2(f)). Additionally, the positive cells of CD34, CD133, and ALDH1 were downregulated by miR-26a-5p overexpression and upregulated by miR-26a-5p inhibition (Figure 2(g)). These finding revealed that miR-26a-5p exerted the antitumor effects in NSCLC by suppressing cell proliferation and cancer stem cell-like properties.

### 3.3. DNMT3A Is a Target of miR-26a-5p

**Figure 3:**
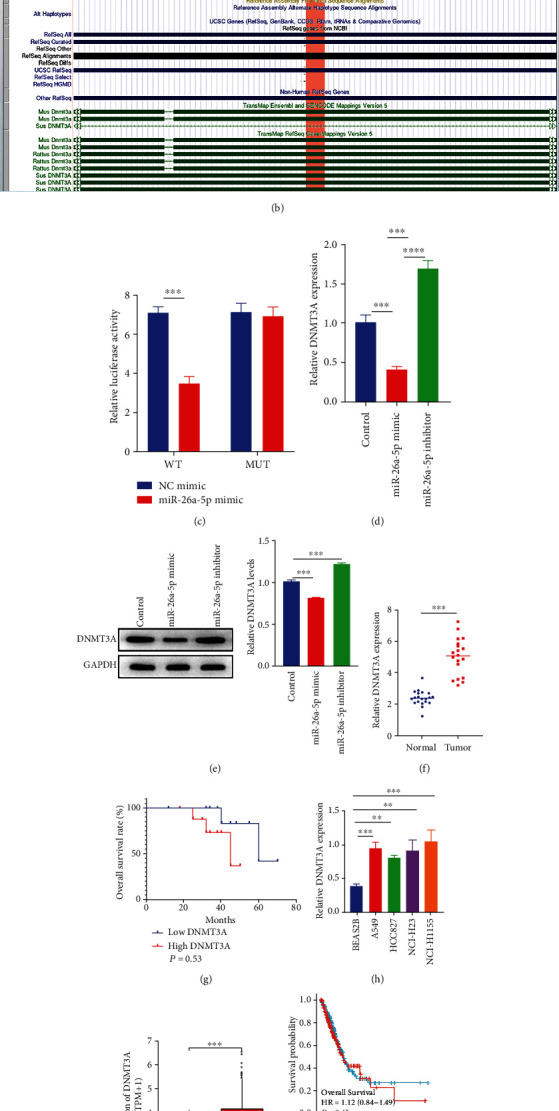
DNMT3A is a target of miR-26a-5p. (a) The binding sites of miR-26a-5p with 3′UTR of DNMT3A were predicated using Starbase database (http://starbase.sysu.edu.cn/index.php). (b) The position of DNMT3A bind with miR-26a-5p was illustrated according to University of California Santa Cruz Genomics Institute (UCSC, http://genome.ucsc.edu/). (c) The dual-luciferase activity of wild/mutant type DNMT3A remodified reporter gene was cotransfected with miR-26a-5p mimic and its negative control. (d, e) The mRNA and protein expressions of DNMT3A were individually examined by qRT-PCR and western blotting after miR-26a-5p upregulation/downregulation. (f) The DNMT3A expression in NSCLC tumor tissues (*N* = 20) and adjacent normal tissues (*N* = 20) was detected by qRT-PCR. (g) Overall survival between high DNMT3A expression group and low DNMT3A expression group was determined by Kaplan-Meier plot using log-rank test. (h) The DNMT3A expression in NSCLC cell lines and BEAS2B was determined using qRT-PCR. (i) The DNMT3A expression in LUAD tumor samples (*N* = 535) and normal samples (*N* = 59) was based on TCGA database. (j) Overall survival between high DNMT3A expression group and low DNMT3A expression group was determined by Kaplan-Meier plot using log-rank test in TCGA database. Control group was used as the blank control. ∗∗∗*P* < 0.001.

Next, we detected the regulatory mechanism of miR-26a-5p in NSCLC. As shown in Figure 3(a), the target genes of miR-26a-5p were predicated using the Starbase database (http://starbase.sysu.edu.cn/index.php). DNMT3A is a speculated target of miR-26a-5p, and the binding sites of miR-26a-5p were identified in coding DNA sequences (208 bp) at chromosome 2: 25,451,332-25,451,539 using University of California Santa Cruz Genomics Institute (UCSC, http://genome.ucsc.edu/) (Figure 3(b)). Then, the binding relationship between miR-26a-5p and DNMT3A was examined using a dual-luciferase activity assay. The results exhibited that the miR-26a-5p remarkably reduced the luciferase activity in the presence of the wild-type DNMT3A pmirGLO vector, whereas the luciferase activity was not reduced in the present of the mutant-type DNMT3A pmirGLO vector (Figure 3(c)). The mRNA and protein levels of DNMT3A were detected after miR-26a-5p was overexpressed and silenced. We found both mRNA and protein levels of DNMT3A were inhibited by overexpressing miR-26a-5p, but increased by silencing miR-26a-5p (Figures 3(d) and 3(e)). Moreover, DNMT3A elevated in NSCLC tumor tissues compared to adjacent normal tissues as well as in NSCLC cell lines compared to BEAS2B (Figures 3(f) and 3(h)). But there is no significant difference between high and low DNMT3A expression groups (Figure 3(g)). The bioinformatic analyzed results supported above findings, which is high expression of DNMT3A in LUAD tumor tissues compared to normal tissues, and no significant difference between high and low DNMT3A expression groups (Figures 3(i) and 3(j)). Above results indicated that DNMT3A is a target of miR-26a-5p, and miR-26a-5p regulated DNMT3A expression both in transcription and posttranscription.

### 3.4. miR-26a-5p Targets DNMT3A to Reduce Global DNA Methylation and Restore SFRP1 Expression

**Figure 4:**
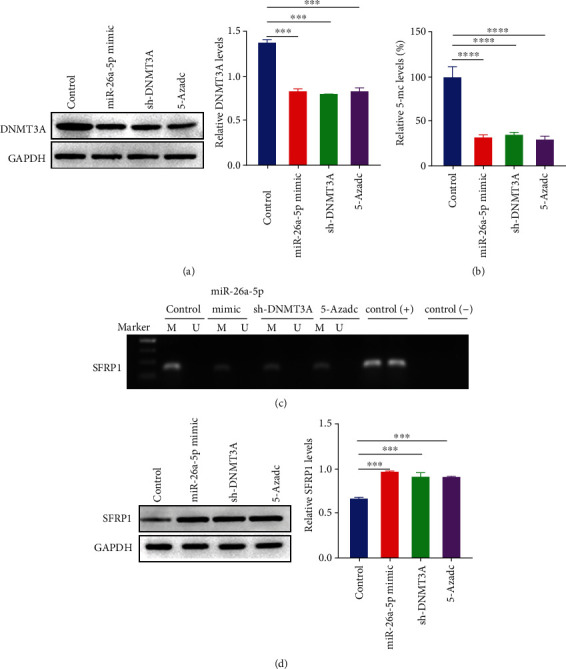
miR-26a-5p targets DNMT3A to reduce global DNA methylation and restore SFRP1 expression. (a)–(d) Protein levels of DNMT3A and SFRP1 were determined by western blotting after miR-26a-5p upregulation, DNMT3A inhibition, and 5-Azadc stimulation. (b) Global DNA methylation was detected by ELISA after miR-26a-5p upregulation, DNMT3A inhibition, and 5-Azadc stimulation. (c) DNA methylation levels of SFRP1 promoter were analyzed by MSP-PCR after miR-26a-5p upregulation, DNMT3A inhibition, and 5-Azadc stimulation. Marker: 1000 bp DNA size marker. Control group was used as the blank control. ∗∗∗*P* < 0.001.

Previous study has demonstrated that SFRP1 is a Wnt antagonist and acts as a tumor suppressor by repressing lung cancer stem-cell like traits, and DNMT3A correlates the epigenetic silencing of SFRP1 gene [30, 31]. Additionally, we found SFRP1 downregulated in NSCLC samples compared to normal samples (Figure S1A-B, S1D-E), as well as SFRP1 downregulated in NSCLC cell lines compared to BEAS2B cells (Figure S1C). Therefore, we focused on the alteration of the DNA methylation in the SFRP1 promoter by DNMT3A. First, we examined the protein levels of DNMT3A after miR-26a-5p overexpression and DNMT3A downregulation by western blotting. The results indicated that the protein levels of DNMT3A were repressed by miR-26a-5p overexpressing, DNMT3A inhibiting, and a DNA methylation inhibitor 5-Aza treating (Figure 4(a)). After that, we found the global DNA methylation levels were reduced by overexpression of miR-26a-5p, knockdown of DNMT3A, and 5-Aza treatment (Figure 4(b)). Furthermore, we detected the methylation status of SFRP1 promoter region using MSP-PCR. The results exhibited that the DNA methylation of SFRP1 promoter was repressed by overexpression of miR-26a-5p and 5-Aza treatment (Figure 4(c)). Moreover, the protein levels of SFRP1 were upregulated by overexpression of miR-26a-5p, knockdown of DNMT3A, and 5-Aza treatment (Figure 4(d)). Our data revealed that miR-26a-5p inhibited DNMT3A to reduce global DNA methylation and restore SFRP1 expression.

### 3.5. miR-26a-5p/DNMT3A/SFRP1 Axis Affects Cell Viability and Stem Cell-Like Phenotype by Regulating Wnt/*β*-Catenin Pathway in NSCLC

**Figure 5:**
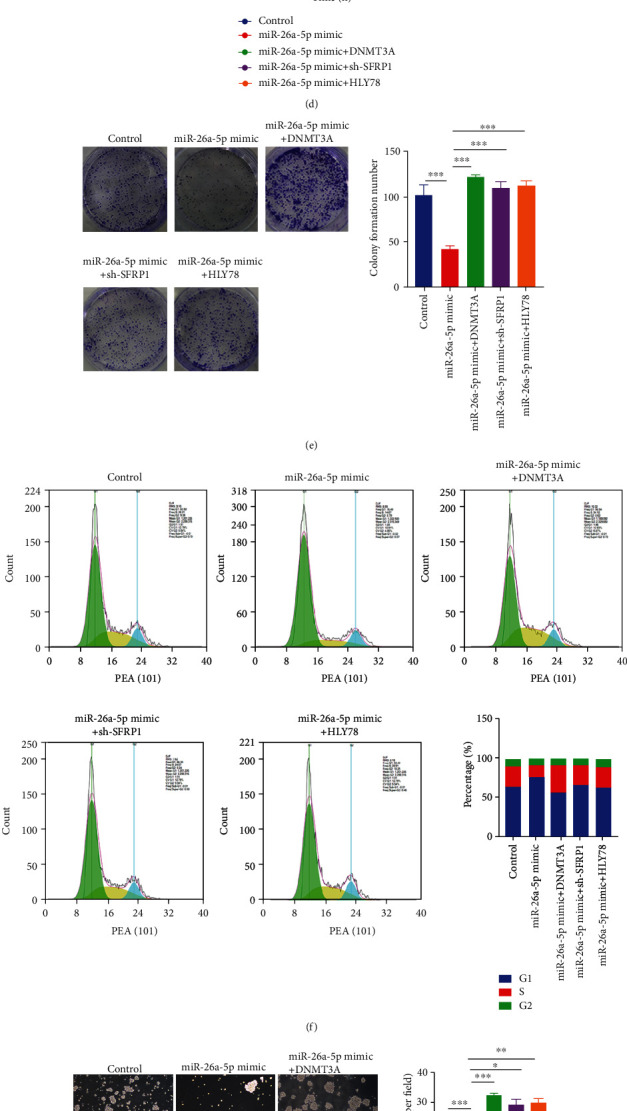
miR-26a-5p/DNMT3A/SFRP1 axis affects cell viability and stem cell-like phenotype by regulating Wnt/*β*-catenin pathway in NSCLC. (a, c, and h) Protein levels of DNMT3A, CCND1, MYC, *β*-catenin, NANOG, OCT4, and SOX2 were determined by western blotting after miR-26a-5p upregulation, miR-26a-5p and DNMT3A coupregulation, miR-26a-5p upregulation and SFRP1 downregulation, and miR-26a-5p upregulation and HLY78 stimulation. (b) The distribution and expression of *β*-catenin in the subcellular fraction after miR-26a-5p upregulation, miR-26a-5p and DNMT3A coupregulation, miR-26a-5p upregulation and SFRP1 downregulation, and miR-26a-5p upregulation and HLY78 stimulation. (d) Cell viability was examined using CCK-8 assay after miR-26a-5p upregulation, miR-26a-5p and DNMT3A coupregulation, miR-26a-5p upregulation and SFRP1 downregulation, and miR-26a-5p upregulation and HLY78 stimulation. (e) Cell colony formation was determined by plate clone assay after miR-26a-5p upregulation, miR-26a-5p and DNMT3A coupregulation, upregulation and SFRP1 downregulation, and miR-26a-5p upregulation and HLY78 stimulation. (f) Cell cycle was detected by FCM after miR-26a-5p upregulation, miR-26a-5p and DNMT3A coupregulation, miR-26a-5p upregulation and SFRP1 downregulation, and miR-26a-5p upregulation and HLY78 stimulation. (g) CSC-like property was detected using sphere formation assay after miR-26a-5p upregulation, miR-26a-5p and DNMT3A coupregulation, miR-26a-5p upregulation and SFRP1 downregulation, and miR-26a-5p upregulation and HLY78 stimulation. (i) CD34, CD133, and ALDH1 positive cells were identified by FCM after miR-26a-5p upregulation, miR-26a-5p and DNMT3A coupregulation, miR-26a-5p upregulation and SFRP1 downregulation, and miR-26a-5p upregulation and HLY78 stimulation. Control group was used as the blank control. ∗*P* < 0.05; ∗∗*P* < 0.01; ∗∗∗*P* < 0.001.

We subsequently examined whether miR-26a-5p/DNMT3A/SFRP1 axis modulated NSCLC cell malignant behaviors by regulating Wnt/*β*-catenin pathway in NSCLC. Western blot results indicated that protein levels of DNMT3A were repressed, and the protein levels of SFRP1 and *β*-actin were increased by miR-26a-5p overexpressing, whereas the effects of miR-26a-5p overexpression on A549 cells were reversed by DNMT3A upregulation, SFRP1 inhibition, and treating with a Wnt/*β*-catenin pathway activator HLY78 (Figures 5(a)–5(c)). The downstream proteins MYC and CCND1 were inhibited by miR-26a-5p overexpression, whereas the effects of miR-26a-5p were harbored by overexpressing of DNMT3A and SFRP1 or treating with HLY78 (Figure 5(c)). Additionally, larger amount of *β*-catenin was aggregated in cytoplasm compared with nucleus by miR-26a-5p overexpressing; however, overexpressing of DNMT3A, knockdown of SFRP1, and treating with HLY78 reversed the effects of miR-26a-5p overexpressing (Figure 5(b)). Cell behavior investigation results revealed that DNMT3A upregulation, SFRP1 inhibition, and treating with HLY78 reversed the inhibitory effects of miR-26a-5p on cell viability, colony formation, and cell cycle (Figures 5(d)–5(f)). The cancer stem cell-like trait analysis also indicated that sphere formation ability, embryonic stem cell markers OCT4, NANOG, and SOX2, and cancer stemness markers CD34, CD133, and ALDH1 expressions were repressed by miR-26a-5p, whereas the effects of miR-26a-5p were reversed by DNMT3A increasing, SFRP1 decreasing, and treating with HLY78 (Figures 5(g)–5(i)). These results indicated that miR-26a-5p/DNMT3A/SFRP1 axis regulated cell viability and stem cell-like phenotype by modulating Wnt/*β*-catenin pathway in NSCLC.

### 3.6. Overexpression of miR-26a-5p Exerts the Antitumor Effects *In Vivo*

**Figure 6:**
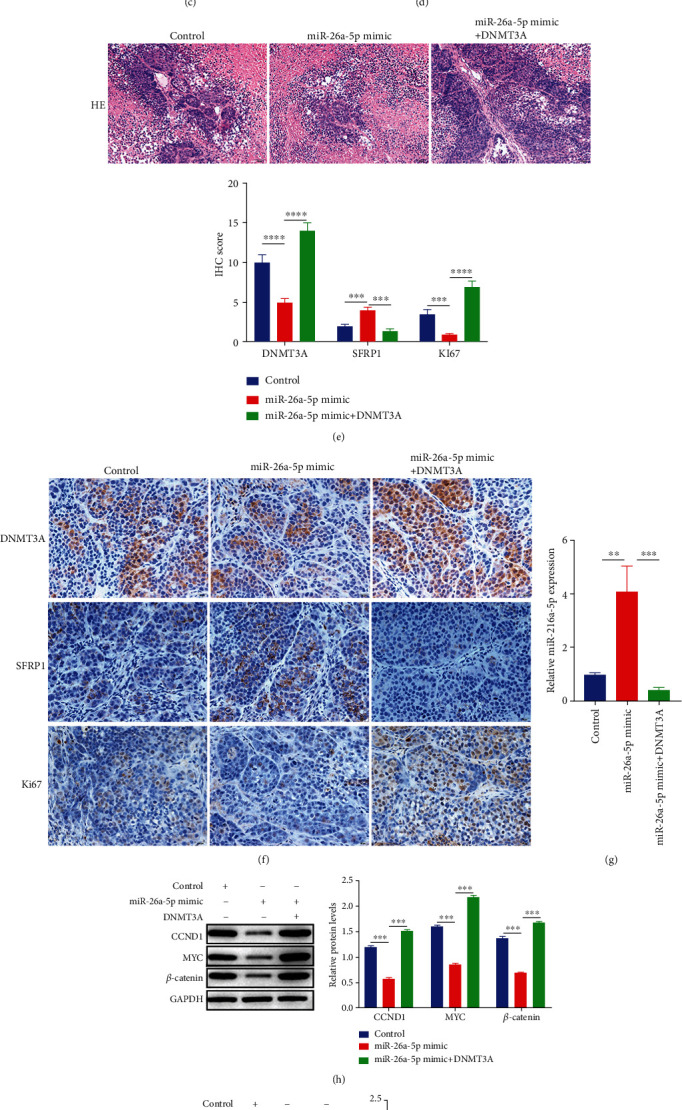
Overexpression of miR-26a-5p exerts the antitumor effects *in vivo*. (a)–(d) The body weight, tumor weight, and volume were detected after miR-26a-5p upregulation, miR-26a-5p, and DNMT3A coupregulation. (e) The pathological change was determined by H&E staining after miR-26a-5p upregulation, miR-26a-5p, and DNMT3A coupregulation. Scale bar = 50 *μ*m. (f) The expression of DNMT3A, SFRP1, and Ki67 was analyzed by IHC after miR-26a-5p upregulation, miR-26a-5p, and DNMT3A coupregulation. Scale bar = 20 *μ*m. (g)–(i) Protein levels of CCND1, MYC, *β*-catenin, NANOG, and SOX2 were examined by western blotting after miR-26a-5p upregulation, miR-26a-5p, and DNMT3A coupregulation. (j) CD34, CD133, and ALDH1 positive cells were identified by FCM after miR-26a-5p upregulation, miR-26a-5p, and DNMT3A coupregulation. Control group was used as the blank control. ∗∗*P* < 0.01; ∗∗∗*P* < 0.001.

Finally, we examined the antitumor effects of miR-26a-5p *in vivo*. As shown in Figures 6(a)–6(d), the tumor size was reduced by miR-26a-5p overexpressing, and the inhibitory effects of miR-26a-5p were reversed by DNMT3A upregulation, whereas no change was found in body weight. The H&E staining results revealed that miR-26a-5p overexpressing inhibited the immune infiltration, but the inhibitory effects of miR-26a-5p were restored by DNMT3A upregulation (Figure 6(e)). IHC results indicated that miR-26a-5p overexpressing reduced the ki67 and DNMT3A positive cells and increased the SFRP1 positive cells, but the effects of miR-26a-5p were stored by DNMT3A upregulation (Figure 6(f)). Moreover, the protein levels of *β*-catenin, MYC, CCND, and stem cell markers (OCT4, NANOG, and SOX2) were reduced by miR-26a-5p overexpressing but reversed by DNMT3A upregulation (Figures 6(h) and 6(i)). The FCM results exhibited the inhibitory effects of miR-26a-5p on cancer stem cell-like properties, and the opposite effects of DNMT3A for miR-26a-5p (Figure 6(j)). These finding revealed that miR-26a-5p exerted the antitumor effects *in vivo*.

## 4. Discussion

**Figure 7:**
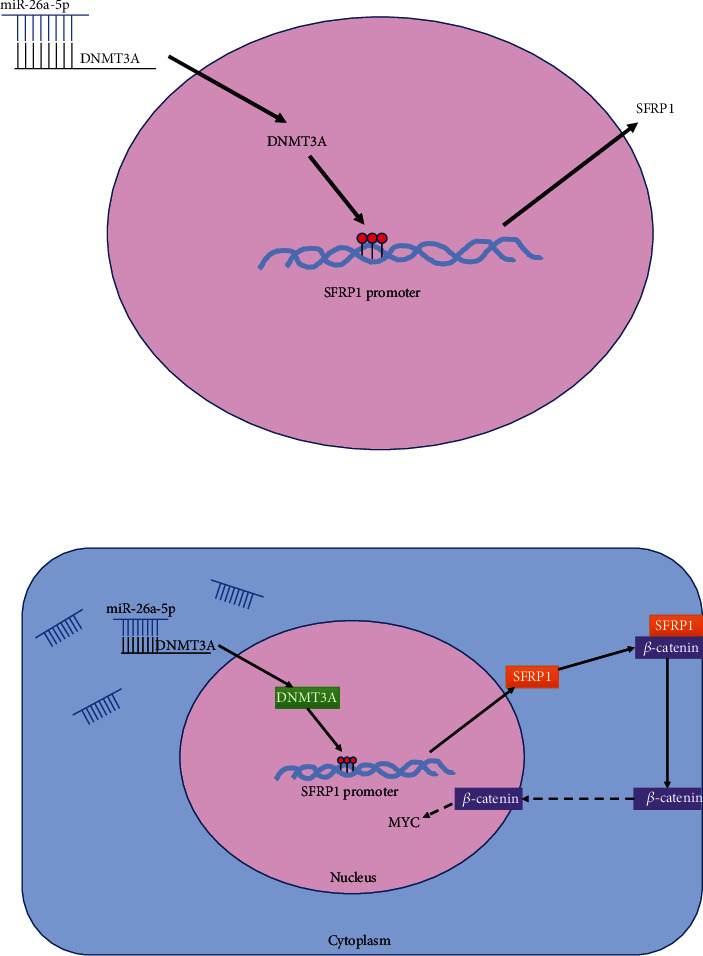
The regulatory mechanism chart of miR-26a-5p/DNMT3A/SFRP1/Wnt/*β*-catenin axis in this study.

In the present study, we found miR-26a-5p exerted antitumor effects on NSCLC by inhibiting cancer stem-cell like properties through downregulating the DNMT3A to increase SFRP1 expression and then increasing SFRP1 inhibited Wnt/*β*-catenin pathway in the regulatory process (Figure 7). Although downregulation of miR-26a-5p in NSCLC, there was no significant distinction of overall survival rate between high miR-26a-5p expression group and low miR-26a-5p expression group. Of interest, low miR-26a-5p expression is positively associated with tumor metastasis and aggressive. Therefore, miR-26a-5p functions as antitumor effects by disrupting tumorigenesis and tumor progression.

Increasing evidences have indicated that the dynamic genic and epigenic alterations affect tumorous cell malignant behaviors and then regulate tumor initiation, metastasis, and chemo/radiotherapy resistance [32]. Since epigenetic alterations have been found to contribute the programing of the stem cells that causes normal stem cells to CSCs with loss of the multilineage differentiation potential and maintain the stem-like properties such as self-renew, proliferation, and invasion of distal tumor sites [33]. Epigenic modification such as DNA methylation and histone methylation reprogram CSCs contributes to multiple cancer initiation, progression, and therapy responses [34–36]. DNA methylation is a pivotal mechanism in cancer that regulate gene expression and cell fate commitment [37, 38]. DNA hypermethylation of the CLDN1 promoter represses lung cancer stem cell-like phenotype and enhances chemotherapeutic efficacy [39]. However, hypomethylation of FOXF1 facilitates cell proliferation, acquires cancer stem properties, and inhibited cell apoptosis to induce cisplatin resistance in NSCLC [36]. Here, we demonstrated DNMT3A-mediated SFRP1 methylation promoted tumor progression in NSCLC.

DNMT3A is a de novo DNA methyltransferase responsible for establishing the early DNA methylation patterns in embryogenesis via de novo DNA methylation on unmethylated CpG sites [40]. Normally, DNMT3A-mediated hypermethylation of promoter on tumor suppressor genes or oncogenes restrains gene expression, which regulate tumor initiation, metastasis, and progression [41]. Numerous studies reveal DNMT3A mutation influences the acute myeloid leukemia progression [42, 43]. Additionally, DNMT3A that has also been discovered acts as a key role in multiple solider cancers. For instance, MYC and DNMT3A-mediated DNA methylations on the miR-200b promoter repress triple negative breast cancer migration, invasion, and cancer stem cell-like properties [44]. Besides, downregulation of DNMT3A remarkably reduces the global DNA methylation and upregulates tumor suppressor CDH1 to repressed NSCLC initiation, development, and stemness [45]. DNMT3A mediates miR-639 promoter methylation to accelerate tumor cell growth, migration, and invasion in liver cancer [46]. DNMT3A also exerts as an oncogene in lung cancer by enhancing DNA methylation of the phosphatase and PTEN to reducing their expression [47]. Above researches indicate that DNMT3A exerts an important role in lung cancer initiation and progression by enhancing DNA methylation on genes; therefore, DNMT3A and its target genes might act the potential therapeutic molecules. Whereas there remain multiple target genes of DNMT3A and underlying regulatory mechanism of DNMT3A unclear. In this study, DNMT3A had been identified as the target of miR-26a-5p and acted as an oncogene role in NSCLC through repressing SFRP1 via DNA methylation modification to suppress Wnt/*β*-catenin pathway.

SFRP1 belongs to the SFRP family which secretes glycoproteins and contains a cysteinerich domain homologous to bind Wnt ligands and antagonize the Wnt signaling pathway [48]. SFRP1 exerts as a tumor suppressor by repressing Wnt/*β*-catenin pathway in multiple tumors, such as breast cancer [49], head and neck squamous cell carcinoma and skin squamous cell carcinoma [50], and ovarian cancer [51]. A large number of evidences indicate that Wnt/*β*-catenin signaling pathway involves in several cellular functions such as organ formation, self-renewal of CSCs, and cell survival [52]. Commonly, Wnt/*β*-catenin pathway includes beta-catenin-dependent and independent signaling pathways, and the beta-catenin-dependent signaling pathway mostly triggered by the LRP-5/6 receptors and Frizzled receptors binding to Wnt ligand, whereas beta-catenin-independent signaling pathway consists of Wnt/Ca^2+^ pathway and planar cell polarity pathway (PCP) [53]. It has been reported that dysregulation of Wnt/*β*-catenin pathway affects tumor initiation, progression, cancer stem cell-like trait acquisition, and drug resistance in NSCLC [54, 55]. Nevertheless, there is a little known about the DNA methylation regulates the Wnt/*β*-catenin pathway in NSCLC. Inspiringly, we found miR-26a-5p regulated DNMT3A expression to remold DNA methylation pattern of SFRP1, therefore, modulated tumor growth and cancer stem cell-like properties via Wnt/*β*-catenin signaling pathway.

Taken together, we indicated that miR-26a-5p played as a tumor suppressor, and DNMT3A acted as an oncogene to repress SFRP1 expression by enhancing DNA methylation. Our finding provided the potential therapeutic targets and molecular mechanism for NSCLC treatment and research.

## Data Availability

Data are available from the corresponding author upon reasonable request.

## Supplementary Materials

Supplementary Materials The supplementary material included the original photographs of the proteic bands. Supplementary Figure 1: original the proteic bands in this manuscript. Supplementary Figure 2: original images of the agarose gel in this manuscript. Table S1: the sequences of oligonucleotides and vector in this study. Table S2; correlation between miR-26a-5p and clinicopathological characteristics in TCGA-LUAD (*n* = 521).
