# Supplementary material for: miR-26a-5p Suppresses Wnt/β-Catenin Signaling Pathway by Inhibiting DNMT3A-Mediated SFRP1 Methylation and Inhibits Cancer Stem Cell-Like Properties of NSCLC
Source: Dis Markers. 2022 Jul 11;2022:7926483. doi: 10.1155/2022/7926483 (PMC9293526; doi:10.1155/2022/7926483)
Supplement: Supplementary Materials — The supplementary material included the original photographs of the proteic bands. Supplementary Figure 1: original the proteic bands in this manuscript. Supplementary Figure 2: original images of the agarose gel in this manuscript. Table S1: the sequences of oligonucleotides and vector in this study. Table S2; correlation between miR-26a-5p and clinicopathological characteristics in TCGA-LUAD (n = 521). [file 7926483.f1.zip › Supplementary Tables (2).docx]

Table S1: The sequences of oligonucleotides and vector in this study.

| Item | Sequences (5’-3’) | Concentration |
| --- | --- | --- |
| MiRNA mimics and inhibitors (sense sequences) | | |
| NC mimic | UCACAACCUCCUAGAAAGAGUAGA | 50 nmol |
| miR-26a-5p mimic | UUCAAGUAAUCCAGGAUAGGCU | 50 nmol |
| NC inhibitor | CAGUACUUUUGUGUAGUACAA | 50 nmol |
| miR-26a-5p inhibitor | AGCCUAUCCUGGAUUACUUGAA | 50 nmol |
| shRNAs (vector Prnat-u6.1/Neo) | | |
| Sh-DNMT3A #1 | CACCATTGGGTAATAGCTCTGAGGCGTTCAAGAGACGCCTCAGAGCTATTACCCAA | 2 μg |
| Sh-DNMT3A #2 | CACCGAATTTGCCGTCTCCGAACCACTTCAAGAGAGTGGTTCGGAGACGGCAAA | 2 μg |
| Sh-DNMT3A #3 | CACCGATGTCCTCAATGTTCCGGCACTTCAAGAGAGTGCCGGAACATTGAGGACA | 2 μg |
| Sh-SFRP1 #1 | CACCGAAGCCGAAGAACTGCATGACCTTCAAGAGAGGTCATGCAGTTCTTCGGC | 2 μg |
| Sh-SFRP1 #1 | CACCGTAGAAGCCGAAGAACTGCATGTTCAAGAGACATGCAGTTCTTCGGCTTCTA | 2 μg |
| Sh-SFRP1 #1 | CACCGTCACACTTAAGCATCTCGGGCTTCAAGAGAGCCCGAGATGCTTAAGTGTGA | 2 μg |

Table S2 Correlation between miR-26a-5p and clinicopathological characteristics in TCGA-LUAD (n=521)

| Characteristics | Low expression of hsa-miR-26a-5p | High expression of hsa-miR-26a-5p | p |
| --- | --- | --- | --- |
| n | 260 | 261 |  |
| T stage, n (%) |  |  | **0.035** |
| T1 | 72 (13.9%) | 101 (19.5%) |  |
| T2 | 146 (28.2%) | 131 (25.3%) |  |
| T3 | 30 (5.8%) | 19 (3.7%) |  |
| T4 | 11 (2.1%) | 8 (1.5%) |  |
| N stage, n (%) |  |  | 0.301 |
| N0 | 162 (32%) | 174 (34.3%) |  |
| N1 | 49 (9.7%) | 46 (9.1%) |  |
| N2 | 42 (8.3%) | 32 (6.3%) |  |
| N3 | 2 (0.4%) | 0 (0%) |  |
| M stage, n (%) |  |  | 1.000 |
| M0 | 184 (49.2%) | 167 (44.7%) |  |
| M1 | 12 (3.2%) | 11 (2.9%) |  |
| Age, meidan (IQR) | 64.5 (59, 72) | 67 (59, 73) | 0.188 |
