# Supplementary figures and images for: miR-26a-5p Suppresses Wnt/β-Catenin Signaling Pathway by Inhibiting DNMT3A-Mediated SFRP1 Methylation and Inhibits Cancer Stem Cell-Like Properties of NSCLC
Source: Dis Markers. 2022 Jul 11;2022:7926483. doi: 10.1155/2022/7926483 (PMC9293526; doi:10.1155/2022/7926483)

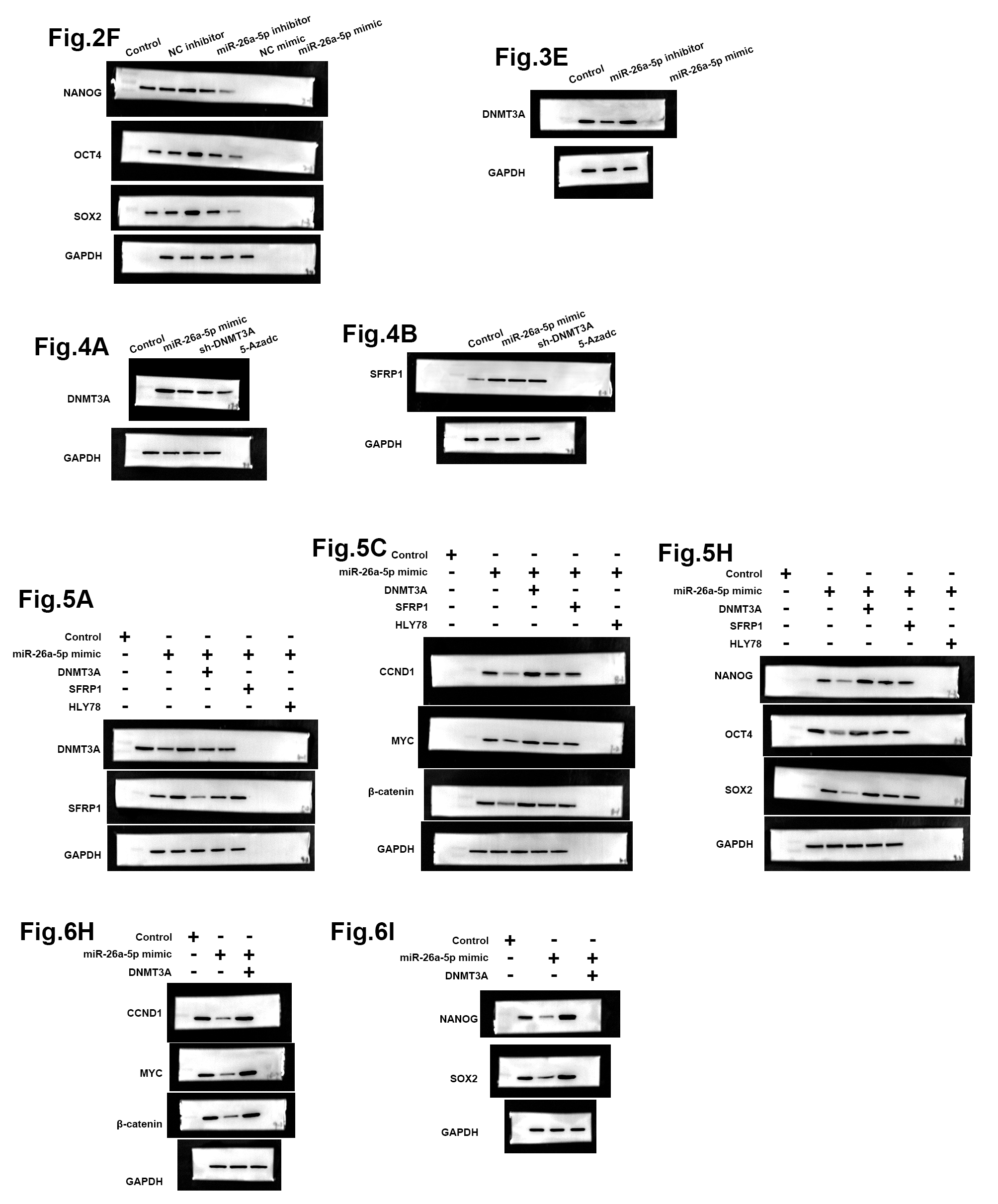

Supplement: Supplementary Materials — The supplementary material included the original photographs of the proteic bands. Supplementary Figure 1: original the proteic bands in this manuscript. Supplementary Figure 2: original images of the agarose gel in this manuscript. Table S1: the sequences of oligonucleotides and vector in this study. Table S2; correlation between miR-26a-5p and clinicopathological characteristics in TCGA-LUAD (n = 521). [file 7926483.f1.zip › Supplementary figures.jpg]
